# Scaling the interactive effects of attractive and repellent odours for insect search behaviour

**DOI:** 10.1038/s41598-019-51834-1

**Published:** 2019-10-25

**Authors:** Thomas A. Verschut, Mikael A. Carlsson, Peter A. Hambäck

**Affiliations:** 1 Department of Ecology, Environment and Plant Sciences, Stockholm University, 106 91 Stockholm, Sweden; 2 Department of Zoology, Stockholm University, 106 91 Stockholm, Sweden

**Keywords:** Behavioural ecology, Entomology

## Abstract

Insects searching for resources are exposed to a complexity of mixed odours, often involving both attractant and repellent substances. Understanding how insects respond to this complexity of cues is crucial for understanding consumer-resource interactions, but also to develop novel tools to control harmful pests. To advance our understanding of insect responses to combinations of attractive and repellent odours, we formulated three qualitative hypotheses; the response-ratio hypothesis, the repellent-threshold hypothesis and the odour-modulation hypothesis. The hypotheses were tested by exposing *Drosophila melanogaster* in a wind tunnel to combinations of vinegar as attractant and four known repellents; benzaldehyde, 1-octen-3-ol, geosmin and phenol. The responses to benzaldehyde, 1-octen-3-ol and geosmin provided support for the response-ratio hypothesis, which assumes that the behavioural response depends on the ratio between attractants and repellents. The response to phenol, rather supported the repellent-threshold hypothesis, where aversion only occurs above a threshold concentration of the repellent due to overshadowing of the attractant. We hypothesize that the different responses may be connected to the localization of receptors, as receptors detecting phenol are located on the maxillary palps whereas receptors detecting the other odorants are located on the antennae.

## Introduction

Before an organism can locate a resource it is usually exposed to a mixture of odours^1,2^. While attractive compounds in a mixture usually evoke movement towards the odour source, repellent compounds can instead impede the organism from moving towards it^3^. A solution to this problem is that the selection of resources generally depends on antagonistic, non-additive interactions among individual odour components^4–6^. Especially for insects, mixtures of attractive and repellent odorants can have interactive effects on the success of finding food^7,8^, mates^9,10^, or oviposition sites^11,12^. Consequently, combinations of attractants and repellents have been used for human benefits to mask human odours from disease vectoring insects^13,14^, to disturb mating of agricultural pest species^15,16^, or to lower the attraction to economically important plants^3^. While all these cases distract insects through the presence of unattractive or masking odorants, the general behavioural principles underlying the combined effect of attractants and repellents on search behaviour are relatively poorly understood. For instance, it is unclear whether attraction to a resource depends on the absolute quantity of individual odorants or on the combined, non-additive effect of the attractants and repellents.

The effects of attractants and repellents depend on how the sensory system detects and processes the different components of an odour mixture. In insects, the olfactory receptors and olfactory receptor neurons (ORN), housed in the sensilla of the peripheral olfactory organs, are the first level where odorants interact^17,18^. Here, repellents may interact with attractants when they activate additional receptors dedicated to repellents^19,20^, or when repellents inhibit the activation of receptors dedicated to attractants^21–23^. Further interactive effects can occur in the antennal lobe, the primary olfactory processing centre in the insect brain, where each functional subunit, or glomerulus, receives signals from specific classes of ORNs^18,24^. At this level, the simultaneous activation of several glomeruli^25,26^, or the concentration dependent activation of specific glomeruli^27–29^, affect how the combination of signals are relayed by projection neurons to higher brain regions: the mushroom body for experience-dependent responses^30–32^, and the lateral horn for innate responses^26,33,34^. While the lateral horn broadly categorizes signals into regions according to their behavioural valence^35–37^, innate attraction and avoidance are processed in parallel pathways which may be responsible for interactive effects between odours^37,38^. Consequently, combined effects of attractants and repellents will depend on the activated receptor^18,24^, the downstream pathway from the ORNs^25,39,40^, and the specific areas in the lateral horn processing the signal^41^.

**Figure 1:**
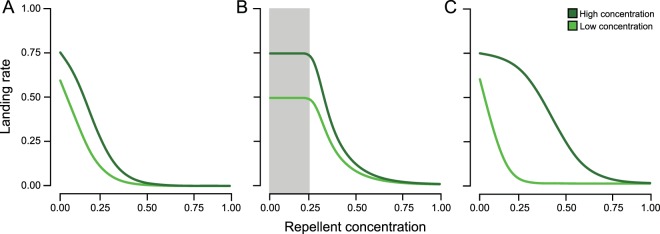
The landing rates on a high (dark green) or low (light green) concentration of attractant is shown in relation to an increasing concentration of a repellent. The response-ratio hypothesis (**A**) predicts an S-shaped curve in which the landing rate is proportionally affected by the increasing concentration of the repellent as by the decreasing concentration of the attractant. In the repellent-threshold hypothesis (**B**) the repellent is only detected above a threshold concentration. The landing rate will not depend on concentration ratios below that threshold (grey area), where the landing rate instead only depends on the concentration of the attractant. This results in a stepwise relationship between the landing rate and the concentration of the repellent. In the odour-modulation hypothesis (**C**) the repellents and attractants act non-additive on the landing rate, resulting in the shape of the dose response of one odorant changing with the absolute concentration of the other odorant.

We have formulated three general hypotheses concerning the relationship between innate responses to mixed odours and the concentrations of attractants and repellents present in a mixture. The first hypothesis, which we coin the response-ratio hypothesis, assumes that the behavioural response depends on the ratio of attractants and repellents. This assumption implies that insect responses are equally affected by the proportional increase in the concentration of repellents as by the proportional decrease in attractants (Fig. 1A). We based the shape of the behavioural response for this hypothesis on two observations. Firstly, there has to be a maximum response at a high ratio of attractant to repellent where all responding insects are attracted. Secondly, electrophysiological measurements on insect antennae have indicated that the probability to detect an odorant increases with the square-root of the odour concentration, rather than proportionally with the concentration of the odour source^42^. Including these two factors in a quantitative relationship results in an S-shaped function in which the maximum response is determined by the concentration of the attractant and where the slope decreases along the increasing concentration of the repellent (Fig. 1A).

The second hypothesis, which we coin the repellent-threshold hypothesis, differs from the response-ratio hypothesis by assuming that repellent odours are only perceived above a threshold concentration and that the behavioural responses will not depend on concentration ratios below that threshold^43,44^. Below the threshold, the response instead only depends on the concentration of the attractant due to overshadowing of the repellent odour (Fig. 1B). The third hypothesis, which we coin the odour-modulation hypothesis, also assumes overshadowing, but through a different mechanism where repellents and attractants have non-additive effects on the behavioural response for all odour concentrations and not only at a threshold. Consequently, the shape of the dose response to one odorant changes with the absolute concentration of the other odorant along the whole concentration gradient. This results in a shift in the steepness of the S-shaped dose response curve of the repellent at different concentrations of the attractant (Fig. 1C). A similar hypothesis was suggested by Schröder and Hilker (2008), who argued that the ability to detect a specific odorant may depend on other odours present in the background.

We translated our hypotheses to statistical models by treating the landing rates of individual insects on a mixture of an attractant and repellent as binomial events. When the landing rates are independently related to the attractant and repellent odorants, as in the response-ratio hypothesis, the expected relationship between the landing rates and the odour concentrations can be modelled in a binomial generalized linear model (GLM). Alternatively, the repellent-threshold hypothesis predicts a more stepwise relationship between the landing rate and the concentration of the repellent odour. Because stepwise relationships result in a nonlinearity between the predictive factors and the odds ratio, such relationships are not properly modelled with a GLM. Therefore, we compared the fit of the GLM to the fit of a binomial generalized additive model (GAM), which allows for non-linear relationships. Finally, the odour-modulation hypothesis deviates from both previous hypotheses by predicting that the relationship between landing rates and one odorant depends on the concentration of the other odorant. This would show up in the GLM as an interactive effect between the attractant and repellent. To further explore the shape of the dose response, we also evaluated the magnitude of the slope. Previous work predicts that the length of the odour plume, and thus the detectability of odorants by the insect’s peripheral olfactory organs, relates to the square-root of the odorant concentration at the odour source^42,46^, resulting in a power relationship with a slope of 0.5. By assuming that plume finding is the rate limiting step in finding an odour source, we expect the slope in the relationship between landing rates and odour concentrations to equal 0.5 in a binomial GLM.

To evaluate the three hypotheses, we quantified the landing rates of *Drosophila melanogaster* Meigen (Diptera: Drosophilidae), as a model organism for olfactory-mediated behaviour, using known attractants and repellents in a wind tunnel. We combined two concentrations of vinegar as an attractant^8^, with five concentrations of benzaldehyde^47^, 1-octen-3-ol^48^, geosmin^19^ and phenol^49^ as repellents. Whereas benzaldehyde and 1-octen-3-ol activate several broadly tuned receptors^47,50,51^, geosmin and phenol are detected by narrowly tuned odorant receptors^19,49^. We evaluated the predictions of our hypotheses in three steps for each combination of odours. First, we tested the odour-modulation hypothesis by testing for the presence of an interactive effect between the repellent and attractant on the landing rates in a GLM. In the absence of a significant interaction, we removed the interaction term and then tested the repellent-threshold hypothesis by determining if a GAM had a better fit than the GLM. In case of a better GAM fit, we modelled the landing rates at low and high concentrations of the repellent separately with GLMs. If these separate analyses would show that the landing rates are affected by the repellent odorant at high concentrations, but not at low concentrations, the behavioural response would fit the repellent-threshold hypothesis. Alternatively, when the GAM did not fit the data better, we concluded that the behavioural response validated the response-ratio hypothesis.

## Material and Methods

### Drosophila rearing

We used 3-day old Canton-S *Drosophila melanogaster* Meigen (Diptera: Drosophilidae) females that were reared under controlled conditions (25 °C, 50% RH, 12:12 L:D) in 28.5 mm × 95 mm rearing vials containing a standard diet of corn syrup (115 mL/L), yeast (26 g/L), soy flour (15 g/L), cornmeal (110 g/L), agar (8.5 g/L) and propionic acid (7 ml/L). We anesthetized the flies with CO_2_ on the day of eclosion and transferred the females to rearing vials containing 1% agar (VWR International AB, Sweden) to starve them for 48 hours prior to the experiments. On the day of the experiments, we transferred individual females into glass tubes (ø 1.5 cm × 10 cm) without anesthetization, closed the glass tubes with humidified cotton balls, and acclimatized the flies to the experimental conditions for at least one hour before testing them.

### Wind tunnel

Our experiments were conducted in a wind tunnel with a 30 cm × 30 cm × 100 cm glass flight section (W x H x L). We used a centrifugal fan to blow air through a filter unit (CamCube CC-0505 - Camfil AB, Sweden) equipped with a pre-filter (Ecopleat 3GPF-F7 - Camfil AB, Sweden), four cylindrical activated carbon filters (Camcarb CG 2600 - Camfil AB, Sweden), and after-filter (Ecopleat 3GPF-F8 - Camfil AB, Sweden), producing a filtered and homogenized airstream of 25 cm/s. To prevent the occurrence of visual disturbances, the wind tunnel was diffusely lit from above by LED-lights set at 7 lux. All the experiments were conducted under controlled environmental conditions (25 ± 1.5 °C, 60 ± 5% RH). We introduced the odours into the flight section through a customized glass Pasteur pipette (ø 1.5 mm) placed in a 30 cm × 30 cm × 20 cm compartment separated from the flight section by a fine metal mesh (pore size 0.63 mm). The Pasteur pipette was placed in an upright position, reaching a height of 7.5 cm, and was connected with Teflon tubing (ø 3 mm) to an odour delivery system that simultaneously blew carbon filtered air (Darco 12–20 mesh; Sigma-Aldrich AB, Sweden) through a 250 ml gas washing bottle containing vinegar, and a customized 50 ml pear-shaped flask containing a repellent. When testing combinations of an attractant and repellent, the airflow from the two bottles were combined by a Y-split to reach the glass pipette at a rate of 0.4 L/min as a single treatment (Model E - Kytola Instruments Oy, Finland). By visualizing the odour plume with titanium tetrachloride (CAS: 7550-45-0 - Merck KGaA, Germany) we found that the plume bent at a height of 15 cm directly above the glass pipette and followed a laminar path reaching a maximum diameter of 15 cm at the downwind end where the flies were released.

**Figure 2:**
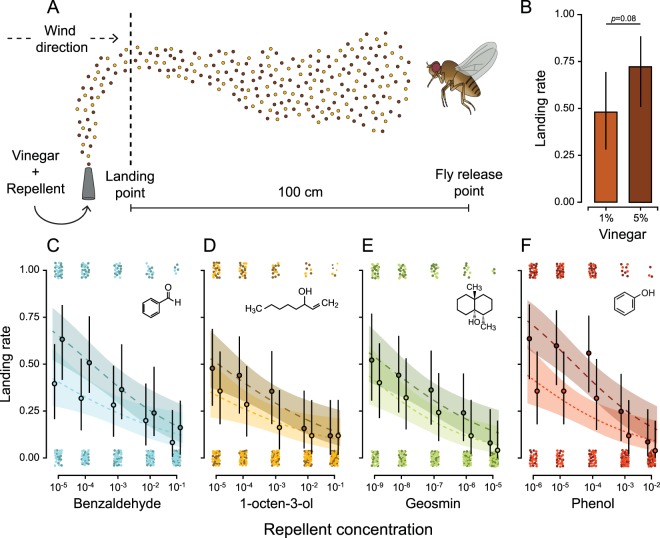
A simplified representation of the wind tunnel assay (not to scale) is given in (**A**). In this example, an individual fly is exposed to an odour plume consisting of a mixture of vinegar (dark brown) and a repellent (yellow). (**B**) The landing rates on 1% and 5% vinegar by themselves. The landing rates on 1% vinegar (lights colours - finely dashed line) and 5% (dark colours - coarsely dashed line) in combination with (**C**) benzaldehyde, (**D**) 1-octen-3-ol, (**E**) geosmin and (**F**) phenol are given with their predicted linear regression lines based on a binomial GLM and their 95% confidence intervals. The actual landing rates on each combination of either 1% (light circles) or 5% (dark circles) vinegar with the repellents are presented with their 95% confidence intervals. The values of landing (1.00) or not landing (0.00) on the odour release point are jittered vertically and horizontally to visualize the binomial data.

We exposed individual flies 100 cm downwind from the odour release point to the different odour treatments by placing the glass tube, in which the flies previously acclimatized, at a release height of 15 cm (Fig. 2A). We firstly tested the attraction rates to 50 ml of 1%, 2.5%, 5% and 10% balsamic vinegar diluted in distilled water (Acetone balsamico di Modena, Urtekram AS, Denmark). We tested these concentrations to ensure that we would not test vinegar concentrations that are inherently repellent. Previous studies showed that *D. melanogaster* has a dose dependent attraction rate to vinegar. While low concentrations of vinegar activate the DM1 and VA2 glomeruli, the additional activation of the DM5 glomerulus at higher concentrations can lead to aversion^7^. While vinegar is composed of a mixture of olfactory compounds^7,52^, we preferred to use vinegar over single compounds due to the low response rate to single attractants in wind tunnels^53^. We found the biggest differences between 1% and 5% vinegar and used these two concentrations as our attractants throughout the rest of the study. Subsequently, we tested the attraction rate to 1% and 5% vinegar in combination with 500 μl of a repellent diluted in Paraffin oil (CAS: 8102-95-1 - Sigma-Aldrich AB, Sweden; see Supplementary Information Fig. S1).

For each repellent we tested five concentrations; 10^−5^ till 10^−1^ of benzaldehyde (CAS No.: 100-52-7, Purity ≥ 99.5% - Sigma-Aldrich AB, Sweden) and 1-octen-3-ol (CAS No.: 3391-86-4, Purity ≥ 98% - Sigma-Aldrich AB, Sweden), 10^−6^ till 10^−2^ of phenol (CAS No.: 108-95-2, Purity ≥ 89% - Sigma-Aldrich AB, Sweden) and 10^−9^ till 10^−5^ of geosmin (CAS No.: 16423-19-1, Purity ≥ 97% - Sigma-Aldrich AB, Sweden). For each treatment, we tested the landing rate of 25 individually released flies which were given 10 minutes to respond to the odour treatment. If the fly would not take off, or land beyond the reach of the odour plume, we would terminate the trial and consider it as non-landing. As a selection criterion for the concentrations we decided that the second highest concentration of the repellent odour should result in at least three landings (out of the 25 tested flies) on the odour release point. This allowed us to determine the shape of the behavioural response to combinations of attractants and repellents irrespectively of *D. melanogaster’s* sensitivity to the specific compound. Finally, we tested the attraction rate to the second highest concentration of each repellent on its own as control treatments (Fig. S1). We used the odours for a maximum of 45 min, after which the odours and glassware were replaced. We considered a fly to be successfully attracted when it landed on the metal mesh, placed in front of the odour delivery compartment, within a 2.5 cm vicinity of where the odour plume bent in the downwind direction. No additional visual markers were used to enhance landing on the mesh, ensuring that the flies only relied on olfactory cues to make their landing decision. After testing ten consecutive flies, we stopped the odour release for approximately 5 minutes to purge the odours from the wind tunnel.

### Statistical analysis

The predictions of our hypotheses were evaluated in three steps for each combination of attractant and repellent. Firstly, to compare the landing rates of *D. melanogaster* on the combination between vinegar and the repellent, we used a generalized linear model (GLM) with a binomial error distribution that included the log vinegar concentration, the log transformed repellent concentration, and an interaction between vinegar and the repellent as explanatory factors. If the interaction between vinegar and the repellent was not significant we removed it though step-wise backward selection based on *χ*^2^ likelihood ratio tests to refute the odour-modulation hypothesis. Secondly, we compared the GLM with a generalized additive model (GAM), that included a smoothing term (k = 3) and an unpenalized upper limit of the degrees of freedom, with the previously explained GLMs excluding the interaction between vinegar and the repellent. The comparison of the GLM with the GAM effectively shows whether the behavioural response is better explained by the response-ratio hypothesis (no difference between GLM and GAM) or the repellent-threshold hypothesis (GAM outcompete the GLM). When the GAM fitted the data better, we modelled the landing rates at low and high concentrations of the repellent separately with GLMs. As our visual inspection of the data suggested a landing threshold around 10^−4^ for phenol, we split the data set at this concentration into a low (10^−6^ till 10^−4^) and high concentration data set (10^−4^ till 10^−2^). Finally, to further explore the shape of the dose response curve in the binomial GLM, we evaluated the magnitude of the slope, for which we expect the parameter describing the relationship between landing rates and odour concentrations to be equal to 0.5. The model assumptions were checked by estimation of overdispersion and inspections of model residuals. All analyses were carried out in R (v. 3.5.0; R Foundation for Statistical Computing, Vienna, AT). The GLMs were performed using lme4^54^, the GAMs with mgcv^55^, and model comparison on *χ*^2^ likelihood ratios with car^56^. The binomial analysis of the landing rates was visualized with ggplot2^57^.

## Results

We first tested the landing rates of individual Canton-S females to 1%, 2.5%, 5% and 10% vinegar to ensure that we would not compare landing rates on vinegar concentrations that may cause repellency (Fig. 2A). We found that the landing rate on 10% vinegar was 12% lower than on 5% vinegar (Fig. S1), suggesting that 10% vinegar may be inherently repellent to *D. melanogaster*. By comparing the other vinegar concentrations, we found the largest difference between 1% and 5% vinegar, with landing rates of 48% and 75% respectively (GLM: *χ*^2^_1,48_ = 3.04, *P* = 0.08; Fig. 2B).

Subsequently, we combined 1% or 5% vinegar with five concentrations of benzaldehyde, 1-octen-3-ol, geosmin or phenol as repellents. For benzaldehyde (Fig. 2C), no interactive effect was found between the vinegar and benzaldehyde concentrations (GLM: *χ*^2^_1,246_ = 0.73, *P* = 0.39), refuting the odour-modulation hypothesis. In the second step, we removed the interaction term and compared the GLM and GAM model fits for the same data. As this comparison showed no evidence for an improved GAM fit (GAM vs. GLM: Δ_*df*_ = 1, *Deviance* = 1.17, *P* = 0.28), we rejected the repellent-threshold hypothesis and instead concluded that the response-ratio hypothesis was best supported for the behavioural response to benzaldehyde. We also found that the parameter for vinegar did not deviate from the predicted value 0.5 (0.46 ± 0.18; Table S1).

For 1-octen-3-ol (Fig. 2D), we found no support for either the odour-modulation hypothesis (GLM interaction: *χ*^2^_1,246_ = 0.16, *P* = 0.69) or the repellent-threshold hypothesis (GAM vs. GLM: Δ_*df*_ = 1, *Deviance* = 0.09, *P* = 0.76), suggesting that the response-ratio hypothesis best supported the behavioural response to 1-octen-3-ol. Furthermore, the parameter value for the relationship between vinegar and the landing rates fell in the range of the predicted value of 0.5 (0.37 ± 0.19, Table S1). For geosmin, we lowered the tested concentrations (Fig. 2E; see methods) to account for *D. melanogaster*’s high sensitivity to this compound^19,53^. We found no significant interaction between geosmin and vinegar (GLM: *χ*^2^_1,246_ = 0.06, *P* = 0.8), and the GAM did not improve the model fit (GAM vs. GLM; Δ_*df*_ = 1, *Deviance* = 1.92, *P* = 0.17), suggesting that the response-ratio best supported the behavioural response to geosmin. The parameter value for the relationship between vinegar and the landing rates also fell in the predicted range of 0.5 (0.36 ± 0.19, Table S1).

**Figure 3:**
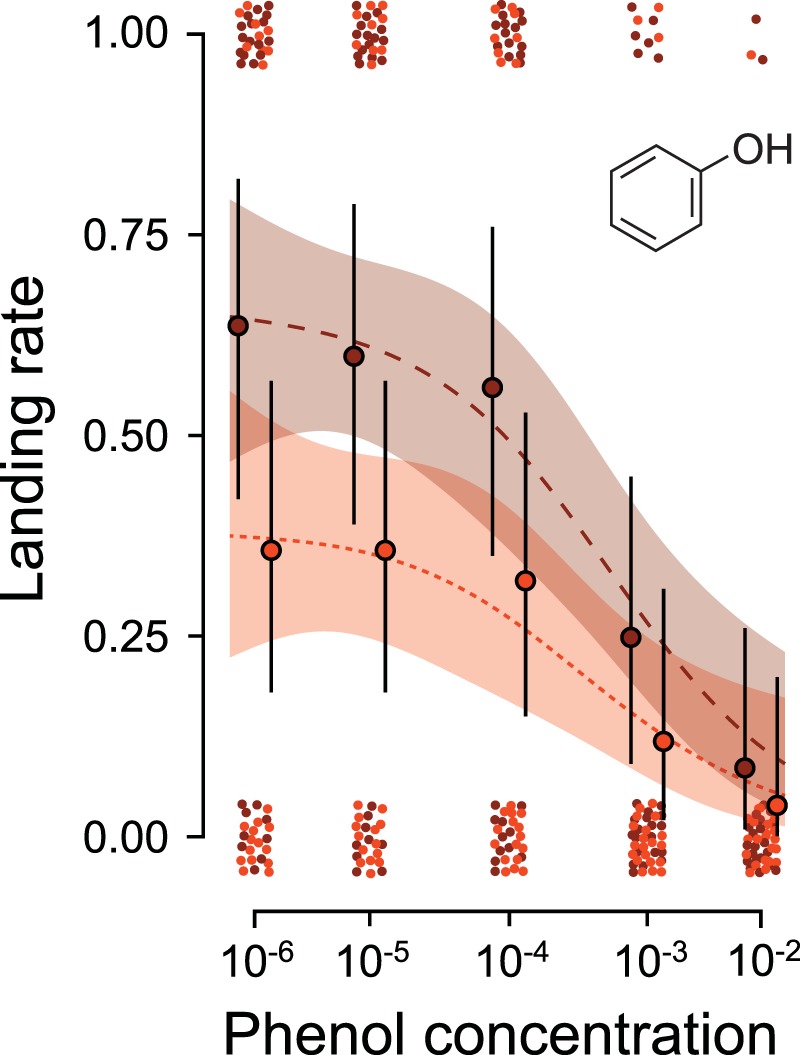
The landing rates on 1% vinegar (lights colours - finely dashed line) and 5% (dark colours - coarse dashed line) in combination with phenol is given with its predicted non-linear regression line and its 95% confidence interval (GAM analysis). The actual landing rates of the combination of either 1% (light circles) or 5% (dark circles) vinegar with phenol is presented with their 95% confidence intervals. The values of landing (1.00) or not landing (0.00) on the odour source are jittered vertically and horizontally to visualize the binomial data.

For phenol, we found no evidence for the odour-modulation hypothesis as there was no interaction between vinegar and phenol on the landing rates (GLM: *χ*^2^_1,246_ = 0.49, *P* = 0.48; Fig. 2F). As the comparison between the GLM and GAM showed an improved GAM fit (GAM vs. GLM; Δ_*df*_ = 1, *Deviance* = 7.61, *P* = 0.005; Fig. 3), the analysis suggested that the response-ratio hypothesis is less relevant for phenol. As the data instead suggested a threshold at the 10^−4^ concentration, we split the data set at this concentration. We then found that the landing rates at low phenol concentrations (10^−6^ till 10^−4^ - Fig. 3) were only affected by the vinegar concentration (GLM: *χ*^2^_1,147_ = 9.78, *P* = 0.002) and not by the phenol concentration (GLM: *χ*^2^_1,147_ = 0.39, *P* = 0.53). For the high phenol concentration (10^−4^ till 10^−2^ - Fig. 3), landing rates were affected by both vinegar (GLM: *χ*^2^_1,147_ = 4.5, *P* = 0.03) and phenol (GLM: *χ*^2^_1,147_ = 22.79, *P* < 0.001), providing support for the repellent-threshold hypothesis. The parameter value for the relationship between vinegar and the landing rates at high phenol concentrations did not deviate from the predicted 0.5 (0.57 ± 0.27).

## Discussion

Understanding how insects integrate attractants and repellents, which presumably provoke opposite behavioural responses, is crucial for understanding consumer-resource interactions. Yet, current research focusses on the identification and practical applications of repellents^58,59^, and less on the quantitative responses by insect to odour mixtures. Therefore, we formulated the response-ratio hypothesis (Fig. 1A), the repellent-threshold hypothesis (Fig. 1B) and the odour-modulation hypothesis (Fig. 1C), as alternative quantitative responses by insects to odour mixtures. We used *Drosophila melanogaster*, as a model organism, to connect the observed behavioural responses in mixed odour environments to our hypotheses, using four known repellents in combination with an attractant. Our experiments provided strong support for the response-ratio hypothesis, which assumes that the behavioural response depends on the ratio between attractants and repellents, for benzaldehyde, 1-octen-3-ol and geosmin. However, the response to phenol rather supported the repellent-threshold hypothesis, where aversion only occurs above a threshold concentration of the repellent compound. Finally, we found no support for the third hypothesis, the odour-modulation hypothesis, which assumes that the tested repellents and attractants have an interactive effect on the behavioural response.

The different responses to the combined odours are likely the result of the ecological relevance and involved sensory pathways, suggesting that further studies of the neuroethological mechanisms are needed to understand insect responses to combined odours. As we did not measure neurological responses in this study, we can only use previous work to speculate about potential mechanism that deserves further investigation. First, for the compounds that supported the response-ratio hypothesis, the most likely hypothesis is that the proportional decrease in landing rates rely on parallel or concentration dependent activation of different glomeruli dedicated to attractants or repellents^7,27^. This process could underlie the proportional decrease observed for the response-ratio hypothesis when it leads to a proportional change in signals with the ratio of the different odorants. This response would depend on the activation of an increasing number of glomeruli that proportionally decreases the activation within the individual glomerulus^27^, resulting in the normalization of the overall activity in the antennal lobe^18,29,60^, or in higher brain centres like the lateral horn^26,33,34^.

While we cannot rule out the involvement of concentration depended activation of glomeruli, we hypothesize that the behavioural response to phenol necessitates a different mechanism. The observation that phenol is only repellent above a certain threshold concentration suggests some type of overshadowing, where vinegar only affects the behavioural response to phenol at low concentrations. Although phenol binds to a narrowly tuned olfactory pathway^49^, we cannot exclusively connect such pathways to the repellent-threshold hypothesis. Considering that geosmin also binds to a narrowly tuned olfactory pathway^19^, a more likely hypothesis concerns the different peripheral binding sites. While phenol binds to receptors on the maxillary palps, the other repellents bind to antennal receptors. Therefore, a potential cause for the response to phenol is that lateral inhibition between glomeruli activated by ORNs on the antenna and maxillary palps may be stronger than lateral inhibition between glomeruli activated by ORNs from the antenna alone. Consequently, the antennal responses may have overshadowed the responses from the maxillary palps below the threshold we observed for phenol (Fig. 3).

While we did not find any evidence for the odour-modulation hypothesis, we expect that this hypothesis occurs when an enhanced contrast between specific odorants reduce the activity of neighbouring glomeruli^18,27,61^. In this case, some odorants should overshadow the signal produced by other odorants present in a mixture in a concentration-dependent manner^40^, rather than normalizing the activity between different neural regions. This alternative type of overshadowing seems to be involved in learned host selection by flower visiting insects^62,63^, where the ability to detect a specific odorant depends on the olfactory background^45^. This finding suggests that the odour-modulation hypothesis may be more applicable for experience based olfactory behaviour rather than the innate behavioural responses tested in our experiments.

It should be noted that translating our results to natural conditions requires additional information on the olfactory cues emitted by different resources (e.g. volatility and complexity of natural mixtures) and the response of insects to variations in odour concentrations. Previous studies have shown that the length of the odour plume increases with the square-root of the odour source concentration^42,46^, resulting in a power relationship with a slope of 0.5, such that a four times higher odour concentration produces an odour plume that is twice as long. Further studies using trapping methods have found the same quantitative relationship between odour concentrations and trapping rates^64^. In this study, we found the same quantitative relationship of 0.5 for the attractant odour, suggesting that the behavioural responses are explained by the probability of finding the odour source, making it likely that detection of repellent odours similarly depends on the length of the odour plume. Although a wealth of studies have indicated possible ways in which different odours are integrated in the neural system^4–6^, we are still at an early stage in understanding how this integration translates to quantitative insect responses to odour mixtures. Moreover, it cannot be excluded that mechanisms not governed by neural processing (such as molecular interactions prior to reception) may, at least partly, play a role in our observed interactions. Instead, our study provides a first glimpse to motivate further research into this area. This information will be essential for the development of models that predict how insects behave in mixed plant communities and for the development of sustainable pest-control strategies^65,66^. We believe that the combination of neuroethological studies, behavioural experiments and quantitative modelling is crucial for understanding the mechanisms underlying difference in the distribution of olfactory oriented insects in field situations.

## Supplementary information

Supplementary information

## Data Availability

The datasets used and/or analysed during the current study are available from the corresponding author on reasonable request.
