# Supplementary material for: Scaling the interactive effects of attractive and repellent odours for insect search behaviour
Source: Sci Rep. 2019 Oct 25;9:15309. doi: 10.1038/s41598-019-51834-1 (PMC6814803; doi:10.1038/s41598-019-51834-1)
Supplement: Supplementary file 1 — Supplementary information [file 41598_2019_51834_MOESM1_ESM.pdf]

Scaling the interactive effects of attractive and repellent odours for insect search behaviour

Scientific Reports

Supplementary information

Author affiliation:
Thomas A. Verschut[1-2], Mikael A. Carlsson[2] and Peter A. Hambäck[1]
1. Department of Ecology, Environment and Plant Sciences, Stockholm University, 106 91 Stockholm, Sweden.
2. Department of Zoology, Stockholm University, 106 91 Stockholm, Sweden.

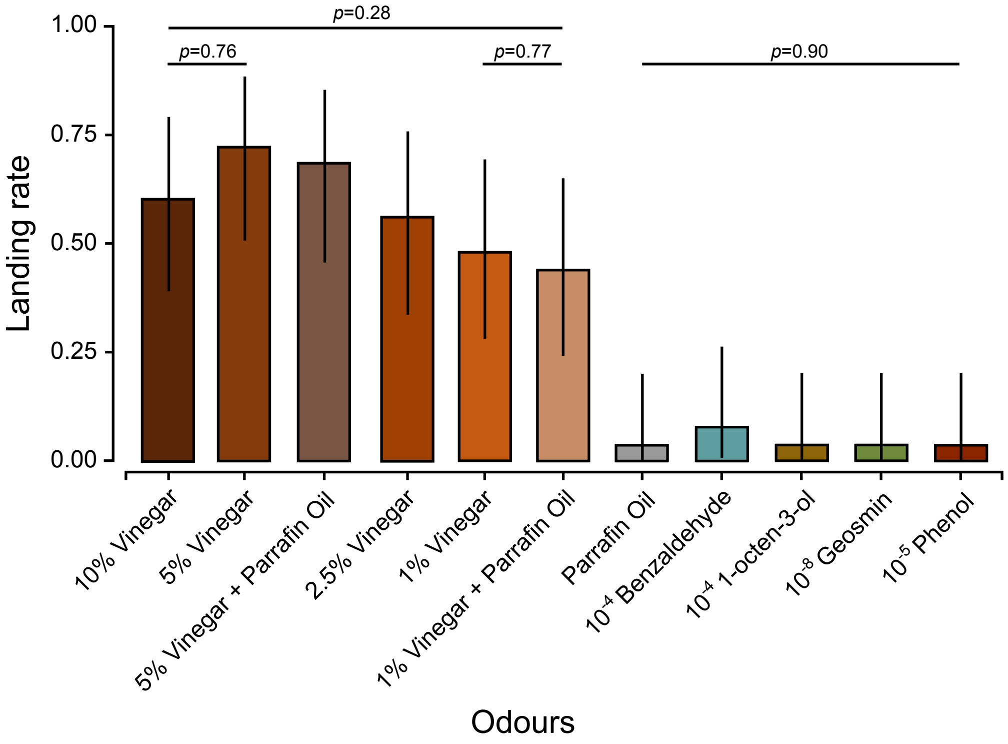

Fig. S1. The landing rates (±95% confidence intervals) of *Drosophila melanogaster* on vinegar and benzaldehyde, 1-octen-3-ol, geosmin and phenol. The landing rates on 1% and 5% vinegar were, respectively, used throughout the study as the low and high vinegar concentration. No effect in combination with 500 µl Paraffin oil (solvent for the repellents) was found for either 1% (GLM: $\chi^2_{1,48}$=0.09, P=0.77) or 5% vinegar (GLM: $\chi^2_{1,48}$=0.08, P=0.76). The second highest concentration of each repellents was tested by itself as a control treatment.

Table S1. Summary of the likelihood ratio tests (χ^2) of the landing rates of *Drosophila melanogaster* on combinations of a low (1%) or high (5%) vinegar concentration with a repellent in the wind tunnel. For the repellents the concentrations used in the experiment are given between brackets. The predicted parameter value is given as the mean ± standard error (i.e. estimate).

		**GLM** (df 1,247)			**GAM** (df 1,246)			**GLM vs. GAM** (Δ_{df}=1)	
**Repellent**	**Factor**	***Estimate ± SE***	χ^2	***P***	***Estimate ± SE***	χ^2	***P***	***Deviance***	***P***
Benzaldehyde (10^{-5} - 10^{-1})	Vinegar	0.46±0.18	6.58	**0.010**	0.46±0.18	6.43	**0.011**	1.17	0.279
	Repellent	-0.22±0.05	26.09	**<0.001**	0.17±0.03	29.72	**<0.001**	-	-
1-Octen-3-ol (10^{-5} - 10^{-1})	Vinegar	0.37±0.19	3.81	0.051	0.37±0.19	3.73	0.053	0.09	0.762
	Repellent	-0.20±0.05	18.29	**<0.001**	-0.21±0.03	38.47	**<0.001**	-	-
Geosmin (10^{-9} - 10^{-5})	Vinegar	0.36±0.19	3.79	0.051	0.36±0.18	3.73	0.054	1.923	0.166
	Repellent	-0.24±0.05	26.49	**<0.001**	0.09±0.02	36.27	**<0.001**	-	-
Phenol (10^{-6} - 10^{-2})	Vinegar	0.61±0.19	11.09	**<0.001**	0.61±0.19	10.81	**0.001**	7.61	**0.005**
	Repellent	-0.27±0.05	34.32	**<0.001**	0.16±0.03	35.13	**<0.001**	-	-
